# Sex‐based differences in swimming performance in 10‐years‐old‐and‐under athletes in short course national competition

**DOI:** 10.1002/ejsc.12237

**Published:** 2024-12-07

**Authors:** Gregory A. Brown, Brandon S. Shaw, Ina Shaw

**Affiliations:** ^1^ Physical Activity and Wellness Laboratory Department of Kinesiology and Sports Science University of Nebraska at Kearney Kearney Nebraska USA; ^2^ School of Sport Rehabilitation and Exercise Sciences University of Essex Colchester Essex UK

**Keywords:** athletic performance, child, female, humans, male, sex characteristics

## Abstract

The need for sex‐segregated youth swimming is debated. A previous report indicates that male swimmers aged 10‐and‐under are 1%–2% faster than females in long course freestyle, butterfly, backstroke, and individual medley (IM), but not breaststroke events. Another report indicates that at age 10 males are 1%–2.5% faster than females in long course freestyle events. However, there are no evaluations for short course competitions. Therefore, the top eight performances for both sexes from the National Club Swimming Association Age Group Championships (a short course meet) for the years 2016–2023 in the 10‐and‐under age group were analyzed. Males were 1.16%–2.63% faster (*p* < 0.05; effect sizes 0.376–0.596) than females in the 50 yards (yd; 45.7 m), 100 yd (91.4 m), and 200 yd (182.9 m) freestyle, 100 yd backstroke, 50 yd breaststroke, 100 yd butterfly, and 100 and 200 yd IM. There were no significant sex‐based differences in the 500 yd (457.2 m) freestyle, 50 yd backstroke, 100 yd breaststroke, or 50 yd butterfly. The individual fastest time for a female was faster than for a male in the 50, 100, and 500 yd freestyle, 50 and 100 yd backstroke, 50 and 100 yd butterfly, and 100 yd IM. Although in eight out of 12 events the individual fastest times were from females, in eight out of 12 events the average male times were significantly faster. The present data suggest that although some exceptional individual 10‐and‐under female swimmers do exist, their performance is not representative of the typical sex‐based differences in swimming performance.

## Highlights

- Males in the 10‐and‐under age group were 1.16%–2.63% significantly faster than females in the 50 yards (yd; 45.7 M), 100 yd (91.4 m), and 200 yd (182.9 m) freestyle, 100 yd backstroke, 50 yd breaststroke, 100 yd butterfly, and 100 and 200 yd individual medley (IM).
- The individual fastest time for a female was faster than for a male in the 50 yd (45.7 m), 100 yd (91.4 m), and 500 yd (457.2 m) freestyle, 50 and 100 yd backstroke, 50 and 100 yd butterfly, and 100 yd IM.
- The performance of the fastest under 10 years old female swimmers may not be representative of the overall sex‐based differences in prepubertal swimming performance.

## 1 INTRODUCTION

The rationale and need for sex‐segregated sports has become a controversial topic in recent years. An argument against sex‐segregated sports is that sex‐segregation in sports propagates outdated gender stereotypes and is an unnecessary holdover of archaic social factors (Leong & Bartlett, 2019). An argument for sex‐segregated sports is that the biological differences between males and females gives males inherent athletic advantages, thus sex‐segregated sports are necessary to protect the safety of female athletes and to provide for fair competition (Hilton & Lundberg, 2021; Hunter et al., 2023). The arguments for and against sex segregated sports become even more contentious when prepubertal children are involved because the focus on sports for these children is often recreation, physical activity, character building, and basic skill development rather than competition (Wells & Arthur‐Banning, 2008). It is also unclear whether there are inherent differences in physical fitness between prepubertal males and females that influence competitive outcomes.

Success in athletic competitions can be influenced by physical fitness. Although it has been reported that there are not sex‐based differences in physical fitness before puberty (Ervin et al., 2014), there are a number of other studies indicating that prepubertal males perform better than prepubertal females on tests of muscular strength and endurance, running speed, and aerobic endurance (Catley & Tomkinson, 2013; Emeljanovas et al., 2020; Tambalis et al., 2016; Tomkinson et al., 2017, 2018). Although enhanced physical fitness can contribute to improved swimming performance (Geladas et al., 2005; Gomez‐Bruton et al., 2016), swimming is a learned skill that requires access to coaching and access to facilities to a much greater extent than do the more innate skills of running, jumping, throwing, and other commonly used activities in physical fitness tests (Olaisen et al., 2018).

In an evaluation of United States of America (USA) Swimming Age Group AAAA long course time standards for all events from 1981 to 2016, Handelsman (2017) indicates that males in the 10‐and‐under age group were ∼1–2% faster than females in freestyle, backstroke, butterfly, and individual medley (IM) events, but there was no sex‐based difference for breaststroke. The data from Handelsman indicates that sex‐based differences for all events increase precipitously from age 11 onwards leading him to conclude that it is not until after the onset of puberty and concomitant increase in testosterone that causes male sex‐based athletic advantages emerge. In the only other evaluation of pre‐pubertal sex‐based differences in competitive swimming performance, Senefeld et al. (2019) evaluated the all‐time top 100 ranked U.S. freestyle swimming performance times for both males and females ages 5–18 years in the 50–1500 m events and concluded that in children under age 10 the top 5 females were faster than the top 5 males, and there were no differences between the 10th–50th place females. However, at age 10, the top 5 males were 2.5% faster than females and the 10th–50th ranked males were 1.0% faster than females. Neither of these evaluations included the short course events, Senefeld only evaluated freestyle events, and neither of these evaluations focused on race times for swimmers at the same swim meet.

Therefore, given the ongoing debate about whether there are sex‐based differences in athletic performance before puberty necessitating sex segregated sport, the limited scholarly evaluations of swimming performance in prepubertal children, and a lack of scholarly evaluation of short course swimming times in children aged 10 and under, an evaluation of competitive swimming performance in short course events in prepubescent children is warranted. The purpose of this project was to determine if there are sex‐based differences in short course swimming events in athletes ages 10‐and‐under (who can reasonably be assumed to be prepubertal) in a National Championship meet. The primary hypothesis was that males swim faster than females in the 10‐and‐under age group in short course national championship competition. A secondary purpose of this project was to compare the differences between finishing order within males and within females to the difference in performance between males and females. The secondary hypothesis was that the percent difference between sexes is greater than the difference within sexes in the 10‐and‐under age group swim in short course national championship competition.

## 2 METHODS

The eight fastest finalist times for males and females for each event each year in the National Club Swimming Association (NCSA, 2024) Age Group Championships for the years 2016–2023 were downloaded in one‐year brackets from the NCSA webpage (https://www.gomotionapp.com/team/recndncsa/page/past‐‐archived‐meet‐results/past‐‐archived‐meet‐results2) between August 30 and December 31, 2023. The NCSA Age Group Championships were selected because of the easily accessible records for past swim meets found on their website, it was desirable to compare athletes swimming at the same swim meets, and the NCSA Age Group Championships is a national event with athletes required to make certified qualifying times to be eligible to compete, therefore this meet should represent some of the best under age 10 swimmers in the USA. The eight fastest finalist times were selected as these should correspond to the finalist heat in NCSA Age Group Championship events. The NCSA Age Group Championship started in 2016 and was not held in 2020 due to the COVID 19 pandemic, so the present data represents all available data in the 10‐and‐under age group to date for this swim meet.

Times for 56 males and 56 females were available for the 50 yd (45.7 m), 100 yd (91.4 m), and 200 yd (182.9 m) freestyle, 50 and 100 yd backstroke, 50 yd butterfly, 100 and 200 yd IM. There were times available for 47 males and 51 females for the 500 yd (457.2 m) freestyle due to there being only two male competitors and three female competitors in 2021, and only five male competitors in 2016. There were times available for 52 males and 56 females for the 50 yd breaststroke due to there being only five male competitors in 2021, and one of those was disqualified. There were times available for 55 males and 55 females for the 100 yd breaststroke due to there being only seven male and seven female competitors in 2021. There were times available for 56 males and 55 females in the 100 yd butterfly due to there being only seven female competitors in 2021.

All procedures for this project accessed public information and did not require ethical review in accordance with the Code of Federal Regulations, 45 CFR 46.102, and the Declaration of Helsinki.

### 2.1 Calculations and Statistics

Data are presented as means ± standard deviation. When necessary, times were converted from minutes:seconds to seconds and all times are reported in seconds. Data for times for all males and females in each event were compared using a two‐tailed two‐sample unequal variance *t*‐test (SigmaStat 4.0, Systat Software). Effect size was calculated using Cohen's *d* when the sample sizes in each sex were the same, and Hedges' *g* when the sample sizes were not. Percent difference between the male and female performances in each event were calculated using the equation described by Millard‐Stafford (Millard‐Stafford et al., 2018).

$$\frac{\text{Male}\;\text{time}-\text{female}\;\text{time}}{\text{Male}\;\text{time}}\times 100$$

For comparison of first versus second, second versus third, third versus fourth, and so on through eighth place, the percent difference between the finishing places in each event were calculated using the equation

$$\frac{\text{Time}\;\text{for}\;\text{second}\;\text{place}-\text{time}\;\text{for}\;\text{first}\;\text{place}}{\text{Time}\;\text{for}\;\text{first}\;\text{place}}\times 100$$

## 3 RESULTS

Males were faster (*p* < 0.05) by 1.69 ± 0.46% than females in the 50 yd (45.7 m), 100 yd (91.4 m), and 200 yd (182.9 m) freestyle, 100 yd backstroke, 50 yd breaststroke, 100 yd butterfly, and 100 and 200 yd IM. There were no significant differences between males and females in 500 yd (4572 m) freestyle, 50 yd backstroke, 100 yd breaststroke, 50 yd butterfly. The individual fastest time for a female was faster than the individuals fastest time for a male in the 50, 100, and 500 yd freestyle, 50 and 100 yd backstroke, 50 and 100 yd butterfly, and 100 yd IM, whereas the individual fastest time for a male was faster than the individual fastest time for a female in the 200 yd freestyle, 50 and 100 yd breaststroke, and 200 yd IM.

### 3.1 Freestyle

**FIGURE 1:**
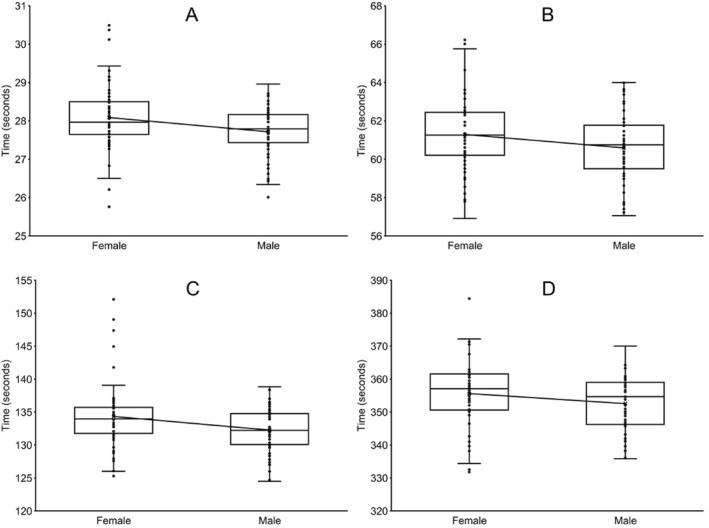
Box plots depicting the finishing times for the 8 finalists in the freestyle events for the 10‐and‐under age group in the National Club Swimming Association Age Group Championships during the years 2016–2023*. The boxes include the first and third quartiles, with the median shown as a solid black line. The whiskers represent the range of data within 1.5* the interquartile range beyond the box boundaries. The solid lines between boxes connect the means. (A). 50 yards (45.7 m) freestyle. *p* = 0.012 for males (*n* = 56) versus females (*n* = 56), effect size (Cohen's *d*) = 0.439 (B). 100 yards (91.4 m) freestyle. *p* = 0.049 for males (*n* = 56) versus females (*n* = 56), effect size (Cohen's *d*) = 0.376 (C). 200 yards (182.9 m) freestyle. *p* = 0.014 for males (*n* = 56) versus females (*n* = 56), effect size (Cohen's *d*) = 0.472 (D). 500 yards (457.2 m) freestyle. *p* = 0.125 for males (*n* = 47) versus females (*n* = 47), effect size (Hedges' *g*) = 0.313. *This swimming meet was not held in 2020 due to the COVID‐19 pandemic.

Males (*n* = 56; 27.72 ± 0.64 s) were 1.34% faster (*p* = 0.012; Cohen's *d* = 0.439) than females (*n* = 56; 28.09 ± 0.87 s) in the 50 yd (45.7 m) freestyle event (Figure 1A). One female (25.76 s) was faster than the fastest male (26.01 s) in the 50 yd freestyle event.

Males (*n* = 56; 60.59 ± 1.78 s) were 1.16% faster (*p* = 0.049; Cohen's *d* = 0.376) than females (*n* = 56; 61.29 ± 1.94 s) in the 100 yd (91.4 m) freestyle event (Figure 1B). One female (56.91 s) was faster than the fastest male (57.06 s) in the 100 yd freestyle event.

Males (*n* = 56; 132.27 ± 3.43 s) were 1.56% faster (*p* = 0.014; Cohen's *d* = 0.472) than females *n* = 56; (134.33 ± 5.12 s) in the 200 yd (182.9 m) freestyle event (Figure [1C]). Two males (124.48 and 124.69 s) were faster than the fastest female (125.3 s) in the 200 yd freestyle event.

There were no significant differences (*p* = 0.125; Hedges' *g* = 0.313) between males (*n* = 47; 352.57 ± 8.37 s) and females (*n* = 51; 355.61 ± 10.79 s) in the 500 yd (457.2 m) freestyle event (Figure 1D). Four females (331.80, 332.08, 332.55, and 334.39 s) were faster than the fastest male (335.86 s) in the 500 yd freestyle event.

### 3.2 Backstroke and breaststroke

**FIGURE 2:**
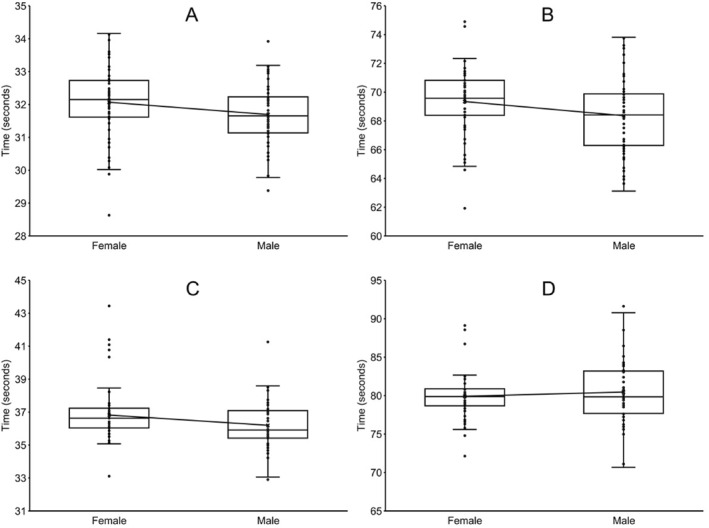
Box plots depicting the finishing times for the eight finalists in the backstroke and breaststroke events for the 10‐and‐under age group in the National Club Swimming Association Age Group Championships during the years 2016–2023*. The boxes include the first and third quartiles, with the median shown as a solid black line. The whiskers represent the range of data within 1.5* the interquartile range beyond the box boundaries. The solid lines between boxes connect the means. (A). 50 yards (45.7 m) backstroke. *p* = 0.055 for males (*n* = 56) versus females (*n* = 56), effect size (Cohen's *d*) = 0.372 (B). 100 yards (91.4 m) backstroke. *p* = 0.040 for males (*n* = 55) versus females (*n* = 55), effect size (Cohen's *d*) = 0.396 (C). 50 yards (45.7 m) breaststroke. *p* = 0.049 for males (*n* = 52) versus females (*n* = 56), effect size (Hedges' *g*) = 0.382 (D). 100 yards (91.4 m) breaststroke. *p* = 0.449 for males (*n* = 55) versus females (*n* = 55), effect size (Cohen's d) = 0.145. *This swimming meet was not held in 2020 due to the COVID‐19 pandemic.

There were no significant differences (*p* = 0.055; Cohen's *d* = 0.372) between males (*n* = 56; 31.69 ± 0.95 s) and females (*n* = 56; 32.07 ± 1.09 s) in the 50 yd (45.7 m) backstroke event (Figure 2A). One female (28.63 s) was faster than the fastest male (29.38 s) in the 50 yd backstroke event.

Males (*n* = 55; 68.36 ± 2.64 s) were 1.44% faster (*p* = 0.004; Cohen's *d* = 0.396) than females (*n* = 55; 69.35 ± 2.35 s) in the 100 yd (91.4 m) freestyle event (Figure 2B). One female (61.92 s) was faster than the fastest male (63.12 s) in the 100 yd backstroke event.

Males (*n* = 52; 36.20 ± 1.45 s) were 1.72% faster (*p* = 0.049; Hedges' *g* = 0.382) than females (*n* = 56; 36.82 ± 1.77 s) in the 50 yd (45.7 m) breaststroke event (Figure 2C). Two males (32.90 and 33.06 s) were faster than the fastest female (33.11 s) in the 50 yd breaststroke event.

There were no significant differences (*p* = 0.449; Cohen's *d* = 0.145) between males (*n* = 55; 80.46 ± 4.12 s) and females (*n* = 55; 79.92 ± 3.28 s) in the 100 yd (91.4 m) breaststroke event (Figure 2D). Two males (70.67 and 71.11 s) were faster than the fastest female (72.13 s) in the 100 yd breaststroke event.

### 3.3 Butterfly and individual medley

**FIGURE 3:**
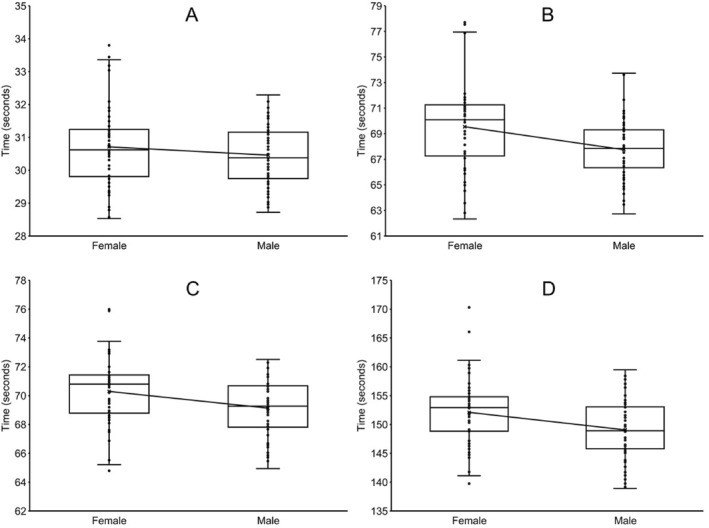
Box plots depicting the finishing times for the eight finalists in the butterfly and individual medley (IM) events for the 10‐and‐under age group in the National Club Swimming Association Age Group Championships during the years 2016–2023*. The boxes include the first and third quartiles, with the median shown as a solid black line. The whiskers represent the range of data within 1.5* the interquartile range beyond the box boundaries. The solid lines between boxes connect the means. (A). 50 yards (45.7 m) butterfly. *p* = 0.267 for males (*n* = 56) versus females (*n* = 56), effect size (Cohen's *d*) = 0.207 (B). 100 yards (91.4 m) butterfly. *p* = 0.002 for males (*n* = 56) versus females (*n* = 55), effect size (Hedges' *g*) = 0.590 (C). 100 yards (91.4 m) IM. *p* = 0.006 for males (*n* = 56) versus females (*n* = 56), effect size (Cohen's *d*) = 0.532 (D). 200 yards (182.9 m) IM. *p* = 0.004 for males (*n* = 56) versus females (*n* = 56), effect size (Cohen's *d*) = 0.550. *This swimming meet was not held in 2020 due to the COVID‐19 pandemic.

There were no significant differences (*p* = 0.267; Cohen's *d* = 0.207) between males (*n* = 56; 30.48 ± 0.95 s) and females (*n* = 56; 30.71 ± 1.25 s) in the 50 yd (45.7 m) butterfly event (Figure 3A). Two females (28.53 and 28.57 s) were faster than the fastest male (28.72 s) in the 50 yd butterfly event.

Males (*n* = 56; 67.77 ± 2.38 s) were 2.63% faster (*p* = 0.002; Hedges' *g* = 0.590) than females (*n* = 55; 69.55 ± 3.55 s) in the 100 yd (91.4 m) butterfly event (Figure 3B). One female (62.33 s) was faster than the fastest male (62.73 s) in the 100 yd butterfly event.

Males (*n* = 56; 69.13 ± 2.00 s) were 1.67% faster (*p* = 0.006; Cohen's *d* = 0.532) than females (*n* = 56; 70.29 ± 2.35 s) in the 100 yd (91.4 m) IM event (Figure 3C). One female (64.79 s) was faster than the fastest male (64.94 s) in the 100 yd IM.

Males (*n* = 56; 149.06 ± 5.31 s) were 2.03% faster (*p* = 0.004; Cohen's *d* = 0.550) than females (*n* = 56; 152.09 ± 5.71 s) in the 200 yd (182.9 m) IM event (Figure 3D). Two males (138.91 and 139.15 s) were faster than the fastest female (139.74 s) in the 200 yd IM event.

### 3.4 Percentage differences between finishing positions

The percentage differences in finishing times between those individuals who would receive medals (i.e., first‐second place, and second‐third place) were 1.46%–1.58% (Table 1). The percentage differences in finishing times between the slowest medalist and the first non‐medalist (i.e., third‐fourth place) were 1.27%–1.33%. In contrast the percentage differences in finishing times between the non‐medalists (i.e., fourth‐fifth, fifth‐sixth, sixth‐seventh, seventh‐eighth) were 0.60%–0.85%.

**TABLE 1:** Percent differences in placing times for the finalists in the 10‐and‐under age group at the National Club Swimming Association Age Group Championships for the years 2016–2023^a^ for all events pooled together.

Place comparison	All swimmers	Females	Males
First–second	1.56 ± 0.54%	1.55 ± 0.62%	1.57 ± 0.48%
Second–third	1.52 ± 0.60%	1.46 ± 0.71%	1.58 ± 0.50%
Third–fourth	1.30 ± 0.45%	1.27 ± 0.39%	1.33 ± 0.53%
Fourth–fifth	0.83 ± 0.32%	0.80 ± 0.28%	0.85 ± 0.37%
Fifth–sixth	0.70 ± 0.29%	0.64 ± 0.20%	0.76 ± 0.36%
Sixth–seventh	0.79 ± 0.32%	0.68 ± 0.26%	0.91 ± 0.34%
Seventh–eighth	0.63 ± 0.22%	0.66 ± 0.26%	0.60 ± 0.18%

*Note*: Data are means ± standard deviation.

^a^ This swim meet was not held in 2020 due to the COVID‐19 pandemic.

## 4 DISCUSSION

The present data indicate that at a national level short course youth swimming meet over seven years of competition when the 8 fastest times in each event for the 10‐and‐under age group are evaluated, males were 1.16%–2.63% significantly faster than females in the 50 yd (45.7), 100 yd (91.4), and 200 yd (182.9 m) freestyle, 100 yd backstroke, 50 yd breaststroke, 100 yd butterfly, and 100 and 200 yd IM. There were no significant sex‐based differences in the 500 yd (457.2 m) freestyle, 50 yd butterfly, 100 yd breaststroke, or 50 yd backstroke (although male times in the 50 yd backstroke were nearly significantly faster at *p* = 0.055). Therefore, because males were faster than females in most, but not all, events, the primary hypothesis that in the 10‐and‐under age group males swim faster than females in short course national championship competition can be partially, but not fully, accepted. The individual fastest time for a female was faster than for a male in the 50, 100, and 500 yd freestyle, 50 and 100 yd backstroke, 50 and 100 yd butterfly, and 100 yd IM, but there were no events in which the female times were significantly faster than male times. Therefore, the outstanding performance of some individual very talented female swimmers at a national level short course youth swimming meet may not be representative of the overall sex‐based differences in swimming performance in children.

In the present data, males were 1.16%–2.63% significantly faster than females in the 50 yd (45.7 m), 100 yd (91.4 m), and 200 yd (182.9 m) freestyle, 100 yd backstroke, 50 yd breaststroke, 100 yd butterfly, and 100 and 200 yd IM, with no sex‐based differences in the 500 yd (457.2 m) freestyle, 50 yd butterfly, 100 yd breaststroke, or 50 yd backstroke. The within sex differences were 1.27%–1.58% for first through fourth place and were 0.63%–0.83% for fourth through eighth place. Therefore, the secondary hypothesis that between sex differences are greater than within sex differences in the 10‐and‐under age group in short course national championship competition can be partially, but not fully, accepted. The data of Handelsman (2017) mostly agree with the present data in that he found that males in the 10‐and‐under age group were ∼1–2% faster than females in long course freestyle, backstroke, butterfly, and IM events, but there were no sex‐based differences for breaststroke. Senefeld et al. (2019) did not break down the data for each event, but indicates that in long course freestyle events the top five ranked females under 10 years old were faster than the top five ranked males under age 10, and there were no differences between the 10th–50th ranked males and females under age 10. However, Senefeld et al. (2019) indicate that at age 10 the top five ranked males were 2.5% faster than the top five ranked females, and the 10th–50th ranked males were 1% faster than the 10th–50th ranked females, which is similar to the present data showing that males in the 10 and under age group were significantly 1.16%–1.56% 50, 100, and 200 yd freestyle events. Although not statistically significant, in the present data for the 500 yd freestyle the times for males were 0.90% faster than the times for females, which closely approximates the overall sex‐based difference in freestyle performance reported by Senefeld et al. (2019). Based on the present, and previous data, males in the 10 and under‐age group swim faster than females of the same age in most, but not all, swimming events and distances.

The USA Swimming records for all‐time short course best performances in the 10‐and‐under age group (as of January 8, 2024), indicate that males are 1.64 ± 0.88% faster than females in the 50 yd (45.7 m), 100 yd (91.4 m), 200 yd (182.9 m), and 500 yd (457.2 m) freestyle, the 50 yd backstroke, the 50 and 100 yd breaststroke, the 50 and 100 yd butterfly, and the 100 and 200 yd IM (USA Swimming, 2024). These records also indicate that the all‐time best performance for a female in the 100 yd backstroke is 1.13% faster than the all‐time performance for a male. The USA Swimming records for all‐time long course best performances in the 10‐and‐under age group (as of January 8, 2024), indicate that males are 1.94% ± 1.25% faster than females in the 50, 100, 200, and 400 m freestyle, the 50 and 100 m backstroke, the 50 m breaststroke, the 100 m butterfly, and the 200 yd IM (USA Swimming, 2024). These same records indicate that all‐time best performances for a female are 1.56 ± 0.17% faster than the all‐time best performance for a male in the 100 m breaststroke and 50 m butterfly. In addition to records of all‐time best performances, USA Swimming produces a chart of motivational times (USA Swimming, 2021) based upon historical performance data that sets times for swimmers in both sexes and every age group in every swimming event, which can then be used to classify the swimmers into progressively faster categories of B, BB, A, AA, AAA, and AAAA. The motivational times can be used by athletes to help them progress into higher levels of competition and can also be used by meet directors to limit participation to swimmers of specific categories. For the 10‐and‐under age group in all categories across 11 long course events and 12 short course events the boys' motivational times are faster than girls' by an average 1.8%, with the exceptions of the 50 yd backstroke where the girls' times are 0.4% faster for the long course and 1.0% faster for the short course. Collectively, the evaluations of Handelsman (2017), Senefeld (Senefeld et al., 2019), the records for all‐time best performances from USA Swimming (USA Swimming, 2024), the motivational times from USA Swimming (USA Swimming, 2021), and the present data all indicate that in the 10‐and‐under age group males swim faster than females in the majority of events. It is particularly common for males in the 10‐and‐under age group to swim faster than females in freestyle and IM events.

In the present and previous data, the sex‐based differences in swimming performance in prepubertal children are between 1.0% and 3.5% with males swimming faster than females in most, but not all, events. Hilton and Lundberg report that adult males swim 11%–13% faster than adult females, but did not differentiate by event (Hilton & Lundberg, 2021). Millard‐Stafford indicates that in US Olympic swimming trials, adult males swim 11.2% ± 1.7% faster than adult females, with greater sex‐based differences in the sprinting rather than endurance events (Millard‐Stafford et al., 2018). Hunter et al. (2023) concurs with Millard‐Stafford that in adults, the sex‐based performance differences are largest in sprint swimming events with the difference being 13.2% for 50 m events and decreasing to 5.7% for 1500 m events. These adult male advantages, particularly in short distance swimming events, are attributed to male having up to twice as much muscular power than females which is facilitated by up to twice as much muscle mass in males (Hunter et al., 2023). In prepubescent children males have approximately 10% more lean body mass than females (McManus & Armstrong, 2011; Staiano & Katzmarzyk, 2012) which corresponds to smaller sex‐based differences in muscle power than in adults (Catley & Tomkinson, 2013; Tomkinson et al., 2018), which helps explain why the sex‐based differences in swimming performance are much smaller in the 10‐and‐under age group than in adults.

In adults, the sex‐based differences in competitive running and swimming performance are similarly in the 5%–13% range for both running and swimming (Hilton & Lundberg, 2021; Hunter et al., 2023; Millard‐Stafford et al., 2018). In contrast, it has recently been reported that prepubescent males run 3%–6% faster in track competition than same aged females (Atkinson et al., 2024; Brown et al., 2024), and yet the present and previous data indicate that the sex‐based difference in swimming performance in prepubescent children is 1%–3.5%.

In comparison to running, the smaller prepubertal sex‐based differences in swimming may be partially explained by noting that swimming is a learned skill (Olaisen et al., 2018). Young swimmers' performance is a multifactorial phenomenon where different factors, such as anthropometry, kinematics, and efficiency, contribute at varying levels (Morais et al., 2017). However, at an early age, there may be individualized variation in kinematic measures, based on training levels (Morais et al., 2017), and varying levels of access to facilities and coaching to a much greater extent than does the innate skill of running (Olaisen et al., 2018). As such, Duke et al. (2023) observed that in addition to sex, school type was a major factor influencing swimming skills since some schools, particularly smaller or less affluent schools, lack aquatic facilities. Those authors also observed that frequency of participation in aquatic activity and prior negative aquatic experiences also contributed to the smaller prepubertal sex‐based differences in swimming performance than other sports. It also appears that more females participate in competitive swimming as children than do males (Senefeld et al., 2019), which can increase the talent pool and level of competition resulting in better performance. It is also speculated that females are more likely to focus solely on swimming and are more likely to enjoy training for competitive swimming (Senefeld et al., 2019), which may also result in better performance. Therefore, it is plausible that increasing the time and type of swimming practice may allow females to overcome some, but not all, of the prepubertal sex‐based anatomical and physiological differences causing sex‐based differences in athletic performance. This evidence suggests that other environmental factors, rather than intrinsic ones (i.e., anthropometrics, such as arm span), are important determinants (Morais et al., 2017) that could allow for some individual under 10 female athletes to excel at such early ages in swimming. In addition, there is evidence that the changes of these determinants over time occur in a nonlinear fashion may account for the reason for only some exceptional performances in individual under 10 female athletes (Morais et al., 2017). Despite these outliers of exceptionally performing individual under 10 female athletes, there is evidence indicating that when male and female children engage in the same amount and intensity of physical activity the males develop greater physical fitness (Dencker et al., 2007; Eiberg et al., 2005), which may predispose males to better athletic performance.

It has been suggested that the prepubertal sex‐based differences in physical fitness and athletic performance are due to males participating in a greater amount of physical activity, higher intensity physical activity, and more sports than do females (Hunter et al., 2023). Supporting this suggestion, Chen et al. (Chen et al., 2018) observed in fifth‐grade children that males spend more time engaged in unstructured physical activity than females, but there was not a sex‐based difference in time spent in organized physical education classes, sports, and dance. Hyde et al. (2020) reported that 60.9% of male children and 54.4% of female children engaged in organized sports whereas Belcher et al. (2010) reported that in children 6 to 11‐years‐old, males participate in more moderate to vigorous physical activity than do females. Collectively, these sources support the notion that prepubertal sex‐based differences in athletic performance can at least be partially explained by higher levels of sport participation and more strenuous physical activity in males than in females. However, in 6 to 7‐year‐old children (Eiberg et al., 2005) and in 8 to 11‐year‐old children (Dencker et al., 2007), the sex‐based differences in maximal oxygen consumption and body composition were not entirely explained by differences in physical activity. Although greater engagement in physical activity and sports is likely to contribute to better sports performance (providing that overtraining syndrome is avoided), and there are some data that indicate male children engage in more sports and physical activity than females, it is not possible to disregard prepubertal sex‐based differences in anatomy and physiology also as causative factors for prepubertal sex‐based differences in athletic performance.

In the present data, evaluating 7 years of national level children's short course swimming competition, the males were 1.16%–2.63% significantly faster than females in the 50 yd (45.7 m), 100 yd (91.4 m), and 200 yd (182.9 m) freestyle, 100 yd backstroke, 50 yd breaststroke, 100 yd butterfly, and 100 and 200 yd IM. Also, in the present data the percentage differences in finishing times between those individuals who would receive medals were 1.46%–1.58%. The percentage difference in finishing times between the non‐medalists was smaller (0.60%–0.85%) than the difference between medalists. Collectively, these data indicate that the time differences between males and females in the 50, 100, and 200 yd freestyle, 100 yd backstroke, 50 yd breaststroke, 100 yd butterfly, and 100 and 200 yd IM are equal to or larger than the time differences between medalists, and are two‐to‐three times larger than the differences between non‐medalists suggesting that the between sex time differences are larger than the within sex differences.

Strengths of the current research are that it represents an evaluation of real‐world competition data for swimming performance in presumably elite prepubertal females and males in short‐course events. The data from the male and female athletes were obtained from the same swim meets which should eliminate possible differences due to seasonal variation (i.e., early, mid, or late season) in performance times. Aside from head‐to‐head competition, these data provide the next best possibility for evaluating sex‐based differences in swimming performance in these athletes and events. The present data also represent, to our knowledge, the first scholarly evaluation of sex‐based differences in short‐course competition in elite prepubertal females and males.

Limitations of the current research are that the present data are based on swim meet records, which offer no insight into the anthropometric characteristics of the athletes other than sex and age. The event records also must be taken at face value, with no knowledge of the accuracy and precision of the timing used for each event. However, the swim meets all occurred under the auspices of NCSA, which has standards for timing and for athlete eligibility. Although these swim meets were labeled as national championships, it is not clear that the best juvenile athletes from across the country are represented among the participants (which is a common challenge in children's sports due to travel costs and family circumstances associated with attending an event outside the immediate local area). However, these above‐mentioned limitations can also be applied to the data evaluated by Handelsman (2017) and Senefeld (Senefeld et al., 2019) and are inherent to the records of best performance for any sport.

Although the current data indicate that prepubertal males swim faster than females in most, but not all, short course events, these data do not provide insight into why these sex‐based differences in performance occur. For example, the lack of sex‐based differences in the 100 yd breaststroke (91.4 m), 500 yd freestyle (457.2 m), and 50 yd butterfly (45.7 m) are intriguing, but difficult to explain. Handelsman (2017) also observed no sex‐based differences in the 100 m breaststroke in the 10‐and‐under age group, whereas there were ∼1% sex‐based differences in all other events. However, the analysis of Handelsman did not include freestyle events over 200 m and only included 100 m for backstroke, breaststroke, and butterfly. The determinants of swimming performance such as swimming economy and energy requirements, propulsive force capacity, and drag force have been extensively studied and could be analyzed to determine why there are and are not sex‐based based differences in prepubertal swimmers (Vilas‐Boas, 2023). For example, females are thought to have buoyancy advantages due to higher percent body fat and are also more fatigue resistant than males (Hunter et al., 2023), which may provide advantages for long duration and energy intense events such as the 500 yd freestyle and 100 yd breaststroke. Furthermore, the better technique of young female swimmers due to more practice (Senefeld et al., 2019) may allow young female swimmers to compensate for the strength and power advantages of males in the 100 yd breaststroke and 500 yd freestyle. But the concept of greater fatigue resistance in young female swimmers does not explain the lack of sex‐based differences in 50 yd butterfly, and conflicts with the presently observed sex‐based differences in the 100 yd butterfly, 100 yd backstroke, 100 and 200 yd freestyle, 100 and 200 yd IM all of which have similar of longer durations than the 100 yd breaststroke. Future research investigating the causative factors for the prepubertal sex‐based differences in swimming performance could evaluate differences in body composition, anthropometrics, time dedicated to swimming specific training, and skill factors that influence swimming performance. Furthermore, the present and previous investigations (Handelsman, 2017; Senefeld et al., 2019) of prepubertal sex‐based differences in swimming performance have been limited to data from the USA. Therefore, evaluations for prepubertal sex‐based differences in swimming performance in other countries would be extremely valuable for informing discussion regarding the necessity (or lack thereof) for sex segregated youth swimming competition. From a practical standpoint, the current research provides coaches and sport scientists with another source of information that can be used to help athletes establish target times to help determine the likelihood of success in a national championship swim meet. The present data can also help inform policy discussions on the necessity of maintaining sex segregated youth swimming competitions.

## 5 CONCLUSION

The present data indicates that prepubertal males swim 1.16%–2.63% significantly faster than females in the 50 yd (45.7), 100 yd (91.4), and 200 yd (182.9 m) freestyle, 100 yd backstroke, 50 yd breaststroke, 100 yd butterfly, and 100 and 200 yd IM, but not in the 500 yd (457.2 m) freestyle, 50 yd butterfly, 100 yd breaststroke, or 50 yd backstroke (although the differences in the 50 yd backstroke approached significance). The present data do not explain why there are sex‐based differences in some, but not all, of these events. Based on the present and previous data indicating that prepubertal males swim faster than prepubertal females in most events, there seems to be little justification to eliminate sex segregated competition in youth swimming. Furthermore, this study serves as a valuable contribution to our comprehension of the intricate interplay between sex differences and swimming performance in prepubertal children. Although further research is imperative to refine and expand upon our current understanding, the findings presented here underscore the significance of considering sex‐specific factors in the assessment of swimming performance in prepubertal children. Acknowledging and investigating these differences not only advances our knowledge in sports science but also holds potential implications for training strategies tailored to individual needs. As we delve deeper into this realm, the multifaceted nature of sex‐related influences on swimming performance in prepubertal children becomes increasingly apparent, allowing for more targeted and effective approaches in the pursuit of athletic safety, inclusion, and excellence.

## CONFLICT OF INTEREST STATEMENT

Dr. Brandon Shaw and Dr. Ina Shaw have no conflicts of interest to declare. Dr. Greg Brown declares that he is currently serving as an expert witness in seven different legal cases in the United States regarding the inclusion of transgender identified males (i.e., trans women) in female sports. No funding that supported this project as part of service as an expert witness was received, his declarations as an expert witness do not include the data presented in this manuscript, and his service as an expert witness is not reliant upon publishing this manuscript.
